# Ginsenoside Rg3 Protects against Diabetic Cardiomyopathy and Promotes Adiponectin Signaling via Activation of PPAR-γ

**DOI:** 10.3390/ijms242316736

**Published:** 2023-11-24

**Authors:** Chenyang Zhang, Huifang Yu, Jingxue Ye, Hongna Tong, Min Wang, Guibo Sun

**Affiliations:** Institute of Medicinal Plant Development, Peking Union Medical College and Chinese Academy of Medical Sciences, Beijing 100193, China; zhangchenyang0120@126.com (C.Z.); 13520717936@163.com (H.Y.); yejingxue2002@126.com (J.Y.); tonghongna@163.com (H.T.)

**Keywords:** ginsenoside, diabetic cardiomyopathy, PPAR-γ, adiponectin, energy homeostasis

## Abstract

Ginsenoside Rg3 extracted from *Panax notoginseng* has therapeutic effects on diabetes and heart diseases. However, the underlying mechanism of ginsenoside Rg3 on diabetic cardiomyopathy (DCM) remains unclear. 24-week-old diabetic db/db mice were treated with ginsenoside Rg3 for 12 weeks, then body weight, serum lipids, adiponectin levels, as well as cardiac function and pathological morphology, were measured. The targets of ginsenoside Rg3 and its regulation of the adiponectin pathway were also evaluated on 3T3-L1 or H9c2 cells. Ginsenoside Rg3 directly bound to PPAR-γ, improving adiponectin secretion and promoting adiponectin signaling. Significantly attenuated overweight, hyperglycemia, and hyperlipidemia, as well as alleviated lipid accumulation and dysfunction in adipose, liver, and heart tissues, were observed in the ginsenoside Rg3-treated group. Ginsenoside Rg3 could be a promising drug targeting PPAR-γ to treat diabetic cardiomyopathy.

## 1. Introduction

The worldwide prevalence of metabolic syndromes, such as obesity and diabetes, is increasing; people with these disorders are more likely to develop heart failure than those without [1]. Abnormal lipid and glucose metabolism and impaired mitochondrial function mainly contribute to the development of DCM [1,2,3]. Although numerous approaches have been reported, glycemic and body weight control remains the first choice for treating DCM [2]. AMPK agonists, SGLT-2 inhibitors, DPP-4 inhibitors, and GLP-1 receptor agonists have been used in patients with diabetes [4]. However, few of them have totally cured or improved prognosis of DCM. Accordingly, it is vital to identify new drugs to specifically treat DCM.

The peroxisome proliferator-activated receptor γ (PPAR-γ) is highly expressed in adipose tissue and contributes significantly to regulating adipocyte function and lipid metabolism [5]. Owing to the potent insulin-sensitizing activity of PPAR-γ, its high-affinity ligands—thiazolidinediones (TZDs)—have been recognized as a potent blood glucose-lowering drug. The heart and other organs can also be protected by TZDs from ischemia/reperfusion injury and shock [6]. However, the currently used TZDs have side effects such as increasing body weight and hepatic toxicity despite their effectiveness in the normalization of blood glucose levels, which renders the discovery of novel ligands highly desirable [7].

Functional foods containing *Panax notoginseng* are widely used globally for their anti-inflammatory, antidiabetic and cardio-protective properties [8,9]. Saponins are the main active ingredient of *Panax notoginseng* to exert numerous pharmacological effects [10]. Our previous research showed that *Panax notoginseng* saponins may protect against diabetic cardiomyopathy and reduce body weight of diabetic mice, however, the mechanism remains unelucidated [11,12]. Ginsenoside Rg3 is a rare ginsenoside extracted from *Panax notoginseng*, and it has broad pharmacological effects such as anticancer, anti-inflammatory, and antiaging properties [13]. In addition, ginsenoside Rg3 protects against metabolic syndromes such as NAFLD, obesity, diabetes, and hypertension [14]. In addition, it may alleviate NAFLD through the PPAR-γ pathway [15]. The saponin also alleviates myocardial infarction and ischemia/reperfusion injury [16]. However, whether ginsenoside Rg3 protects against diabetes mellitus by acting on PPAR-γ without increasing body weight and exacerbating hepatic toxicity remains to be elucidated, as the role of ginsenoside Rg3 in DCM remains largely unknown.

## 2. Results

### 2.1. Ginsenoside Rg3 Alleviated Insulin Resistance and Lowered Serum Lipid Levels in db/db Mice

**Figure 1:**
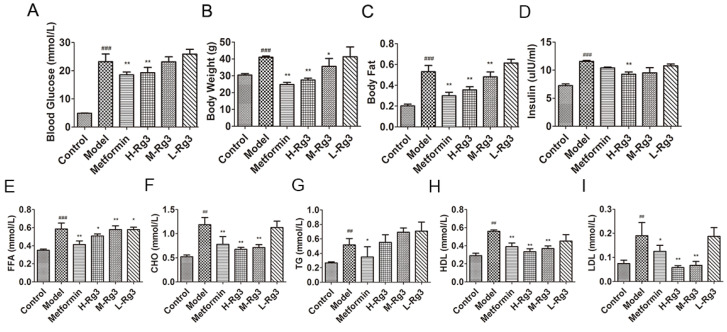
Ginsenoside Rg3 reduced lipid accumulation in diabetic mice. (**A**) Blood glucose level; (**B**) body weight; and (**C**) body fat level of 36-week-old mice after 12 weeks of treatment. (**D**) Serum insulin; (**E**) FFA; (**F**) CHO; (**G**) TG; (**H**) HDL; and (**I**) LDL in indicated groups. Data are expressed as the mean ± SEM (*n* = 3–6). * *p* < 0.05 or ** *p* < 0.01 vs. model group; ^##^ *p* < 0.01 or ^###^ *p* < 0.001 vs. the control. H-Rg3, 100 mg/kg/day; M-Rg3, 50 mg/kg/day; L-Rg3, 25 mg/kg/day.

As shown in Figure 1A–C, the blood glucose levels, body weight, and body fat in the model group were all significantly increased (*p* < 0.001) compared to the control. The blood glucose, body weight, and body fat were significantly reduced (*p* < 0.01) by metformin. H-Rg3 (100 mg/kg) also improved all of these parameters (*p* < 0.01), while M-Rg3 (50 mg/kg) only improved body weight and body fat (*p* < 0.05), L-Rg3 (25 mg/kg) did not improve any of them. Increased serum insulin and free fatty acid levels in diabetic mice were improved after H-Rg3 treatment (Figure 1D,E, *p* < 0.05), while M-Rg3 and L-Rg3 only alleviated levels of free fatty acid (*p* < 0.05). Increased levels of serum lipids such as CHO, TG, HDL, and LDL in the model group were all reduced by metformin (Figure 1F–I, *p* < 0.05). While H-Rg3 and M-Rg3 improved CHO, HDL, and LDL levels (*p* < 0.01), L-Rg3 did not yield any improvement. 

### 2.2. Ginsenoside Rg3 Improved Adiponectin Secretion and Function of Adipose Tissue

**Figure 2:**
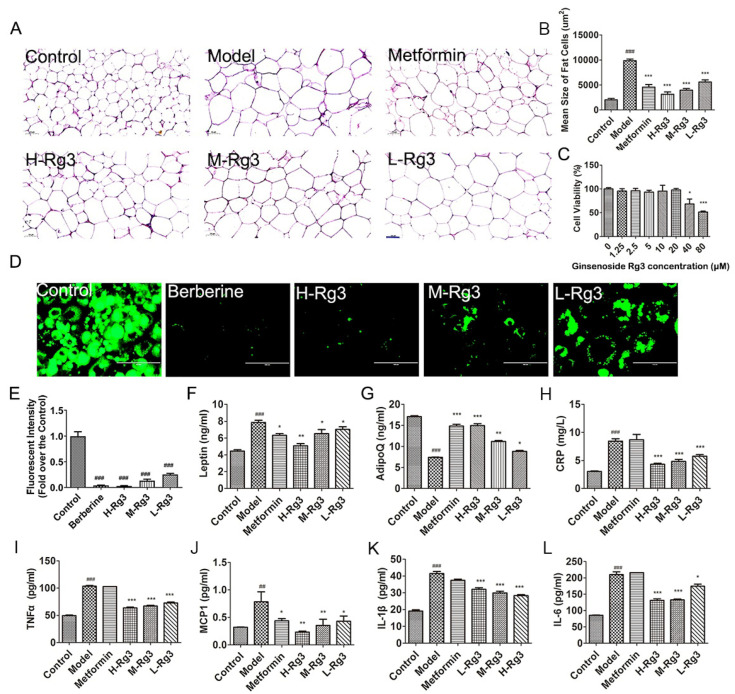
Ginsenoside Rg3 improved secretion of adiponectin and adipose function of diabetic mice. (**A**) Representative images of HE staining of adipose tissue and (**B**) quantification of adipocyte area. Scale bar, 50 μm. Magnification, 200×. (**C**) CCK-8 assay showing 3T3-L1 cell viability treated using different concentrations of ginsenoside Rg3. (**D**) BODIPY staining images showing lipid accumulation in 3T3-L1 cells and (**E**) quantification of green fluorescence in different groups. Scale bar, 200 μm. Magnification, 20×. H-Rg3, 20 μM; M-Rg3, 10 μM; L-Rg3, 5 μM. (**F**) Leptin; (**G**) adiponectin; (**H**) CRP; (**I**) TNFα; (**J**) MCP1; (**K**) IL-1β; and (**L**) IL-6 levels in sera of different groups. Data are expressed as the mean ± SEM (*n* = 3–6). * *p* < 0.05 or ** *p* < 0.01 or *** *p* < 0.001 vs. model group; ^##^ *p* < 0.01 or ^###^ *p* < 0.001 vs. the control. H-Rg3, 100 mg/kg/day; M-Rg3, 50 mg/kg/day; L-Rg3, 25 mg/kg/day.

As shown in Figure 2A,B, metformin and ginsenoside Rg3 administration significantly reduced the size of adipocytes (*p* < 0.001), indicating metformin and ginsenoside Rg3 ameliorated lipid accumulation in diabetic mice. Additionally, berberine acted as a positive control in cellular assay in that it has been reported to significantly reduce lipid levels [17]. The CCK-8 assay showed that 5 μM ginsenoside Rg3 (L-Rg3), 10 μM ginsenoside Rg3 (M-Rg3), and 20 μM ginsenoside Rg3 (H-Rg3) were non-toxic and could be used for further experiments (Figure 2C). BODIPY staining showed that berberine and ginsenoside Rg3 significantly decreased lipid accumulation in mature adipocytes (Figure 2D,E, *p* < 0.001). In addition, metformin and ginsenoside Rg3 improved the changes in adipocytokines such as leptin and adiponectin levels (Figure 2F,G, *p* < 0.05). After treatment with ginsenoside Rg3, proinflammatory cytokine levels in diabetic mice significantly decreased, including CRP, MCP1, TNF-α, IL-1β, and IL-6 (Figure 2H–L, *p* < 0.05), while metformin only decreased MCP1 (*p* < 0.05). 

### 2.3. Ginsenoside Rg3 Reduced Liver Lipid Accumulation and Alleviated HEPATIC Dysfunction

Metformin and ginsenoside Rg3 supplementation significantly improved enlarged and vacuolar liver cells in diabetic mice (Figure S1A). Moreover, ALT and AST levels also markedly decreased by H-Rg3 treatment (Figure S1B,C, *p* < 0.05), whereas serum ALT levels were improved by metformin, M-Rg3, and L-Rg3 in diabetic mice (*p* < 0.05). The lipid accumulation in the model group was also markedly improved by metformin as well as ginsenoside Rg3 (Figure S1D,E, *p* < 0.05). While ginsenoside Rg3 improved serum CAT, GSH, MDA, and SOD levels in diabetic mice, metformin improved none of these parameters (Figure S1F–I, *p* < 0.05). 

### 2.4. Ginsenoside Rg3 Alleviated Cardiac Dysfunction in Diabetic Mice

**Figure 3:**
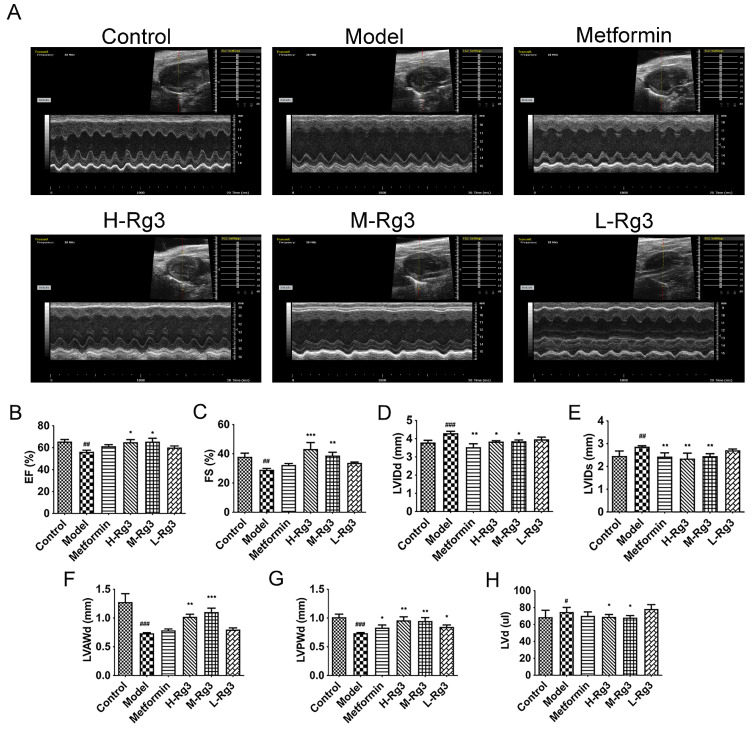
Ginsenoside Rg3 ameliorated cardiac dysfunction in diabetic mice. (**A**) Representative images of echocardiography from mice after 12 weeks of treatment. Statistical analyses of (**B**) EF; (**C**) FS; (**D**) LVIDd; (**E**) LVIDs; (**F**) LVAWd; (**G**) LVPWd; and (**H**) LVd in indicated groups. Data are expressed as the mean ± SEM (*n* = 5). * *p* < 0.05 or ** *p* < 0.01 or *** *p* < 0.001 vs. model group; ^#^ *p* < 0.05 or ^##^ *p* < 0.01 or ^###^ *p* < 0.001 vs. the control. H-Rg3, 100 mg/kg/day; M-Rg3, 50 mg/kg/day; L-Rg3, 25 mg/kg/day; EF, left ventricular ejection fraction; FS, fractional shortening; LVIDd, left ventricular internal diameter in diastolic end; LVIDs, left ventricular internal diameter in systolic end; LVAWd, left ventricular anterior wall thickness in diastolic end; LVPWd, left ventricular posterior wall thickness in diastolic end; LVd, left ventricular volume in diastolic end.

The decreased EF and FS in diabetic mice were significantly improved by H-Rg3 as well as M-Rg3, indicating improved systolic function (Figure 3A–C, *p* < 0.05). In addition, H-Rg3 and M-Rg3 ameliorated thinner LVAWd and LVPWd, as well as decreased LVIDd, LVIDs, and LV volume in diabetic mice, indicating ameliorated ventricular wall thickness and ventricular dilation. However, metformin and L-Rg3 only increased LVPWd in diabetic mice (Figure 3D–H, *p* < 0.05). All data above indicate that ginsenoside Rg3 treatment could improve cardiac function and protect against heart failure in diabetic mice.

### 2.5. Ginsenoside Rg3 Attenuated Cardiac Hypertrophy, Fibrosis, and Apoptosis in Diabetic Mice

**Figure 4:**
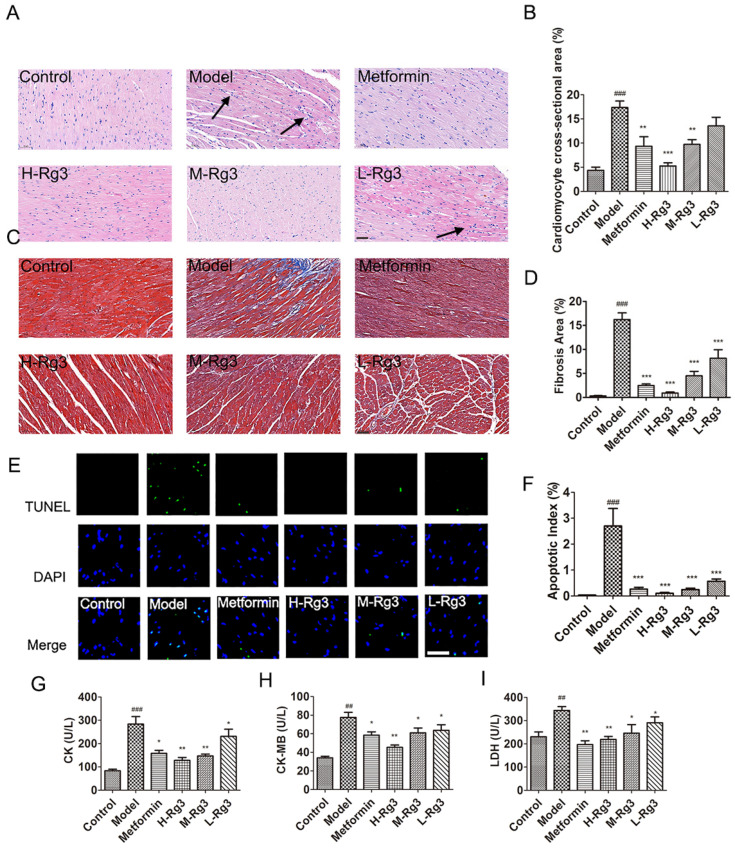
Ginsenoside Rg3 reduced myocardial hypertrophy, fibrosis, and apoptosis in diabetic mice. (**A**) Representative images of hematoxylin–eosin (HE) staining and (**B**) cross-sectional areas in indicated groups. Black arrows indicate inflammatory cell infiltration. Scale bar, 50 μm. Magnification, 200×. (**C**) Representative images of Masson staining and (**D**) statistical analyses of fibrotic ratios in different groups. Scale bar, 50 μm. Magnification, 200×. (**E**) Representative TUNEL staining images and (**F**) statistical analyses of cardiomyocyte apoptotic index in five groups. Scale bar, 50 μm. Magnification, 800×. Serum (**G**) CK; (**H**) CK-MB; and (**I**) LDH in indicated groups. Data are expressed as the mean ± SEM (*n* = 3–6). * *p* < 0.05 or ** *p* < 0.01 or *** *p* < 0.001 vs. model group; ^##^ *p* < 0.01 or ^###^ *p* < 0.001 vs. the control. H-Rg3, 100 mg/kg/day; M-Rg3, 50 mg/kg/day; L-Rg3, 25 mg/kg/day.

It was shown that ginsenoside Rg3 and metformin markedly attenuated the increased cross-sectional areas and fibrosis in diabetic heart by HE and Masson’s staining (Figure 4A–D, *p* < 0.01). Additionally, ginsenoside Rg3 and metformin could also alleviate increased inflammatory cell infiltration and destroyed cardiac morphology in diabetic heart TUNEL staining showed ginsenoside Rg3 and metformin treatment alleviated apoptosis of cardiomyocytes in diabetic mice (Figure 4E,F, *p* < 0.001). Furthermore, H-Rg3 decreased serum CK, CK-MB, and LDH levels in diabetic mice, which reflected alleviated cardiac dysfunction (Figure 4G–I, *p* < 0.05). However, metformin, M-Rg3, and L-Rg3 only improved CK and LDH levels.

### 2.6. Ginsenoside Rg3 Lowered Lipid Accumulation in Diabetic Heart through Improving Lipid and Glucose Metabolism

**Figure 5:**
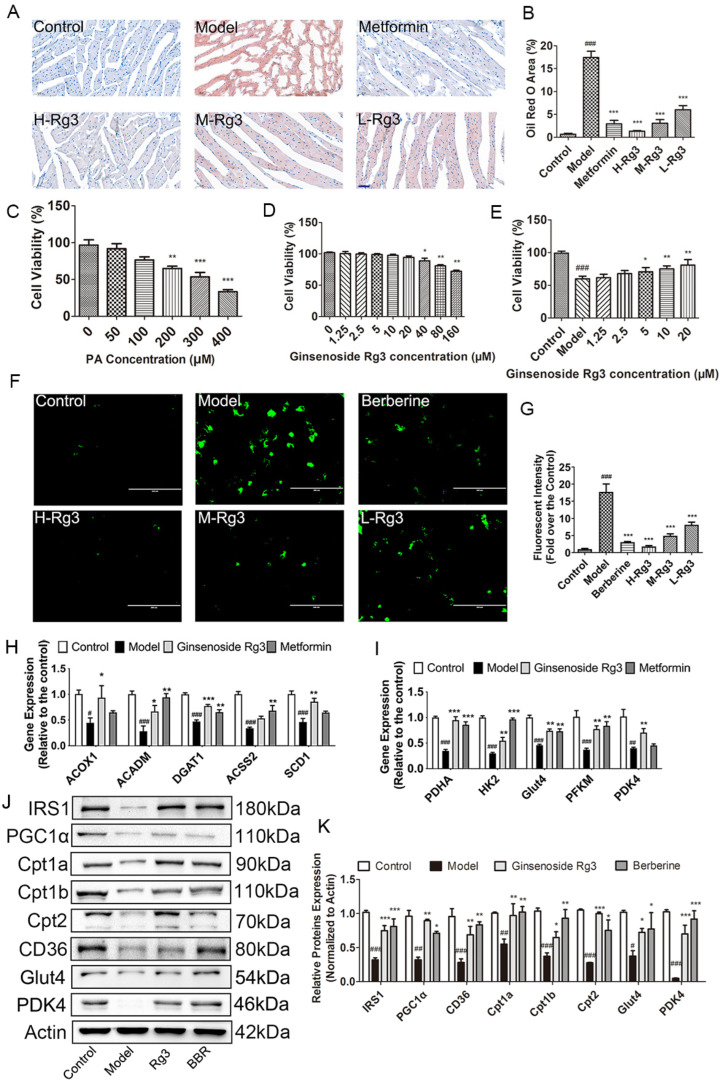
Ginsenoside Rg3 reduced lipid accumulation by promoting lipid and glucose metabolism in diabetic heart. (**A**) Representative images of Oil Red O staining and (**B**) quantitative analysis of Oil Red O staining in six groups. H-Rg3, 100 mg/kg/day; M-Rg3, 50 mg/kg/day; L-Rg3, 25 mg/kg/day. Scale bar, 50 μm. Magnification, 200×. CCK-8 assay showing H9c2 cell viability by (**C**) different concentrations of PA; (**D**) different concentrations of ginsenoside Rg3; and (**E**) pretreatment with different concentrations of ginsenoside Rg3 before adding PA. (**F**) Representative images of BODIPY staining (**G**) and quantitative analysis of green fluorescence in H9c2 cells. Scale bar, 100 μm. Magnification, 400×; H-Rg3, 20 μM; M-Rg3, 10 μM; L-Rg3, 5 μM. Transcription levels of genes involved in (**H**) lipid metabolism and (**I**) glucose metabolism in four indicated groups. (**J**) Representative blot images of proteins related to lipid metabolism and glucose metabolism. (**K**) Quantitative analysis of protein levels. Data are expressed as the mean ± SEM (*n* = 3–6). * *p* < 0.05 or ** *p* < 0.01 or *** *p* < 0.001 vs. model group; ^###^ *p* < 0.001 or ^##^ *p* < 0.01or ^#^ *p* < 0.05 vs. the control.

In vivo study revealed that ginsenoside Rg3 and metformin reduced cardiac lipid droplets in diabetic mice (Figure 5A,B, *p* < 0.001). In vitro assays demonstrated that H9c2 cell viability was reduced to 60% after 200 μM PA treatment for 24 h, and pretreatment with 5 μM ginsenoside Rg3 (L-Rg3), 10 μM ginsenoside Rg3 (M-Rg3), and 20 μM ginsenoside Rg3 (H-Rg3) significantly increased cell viability (Figure 5C–E, *p* < 0.01). It was also demonstrated that ginsenoside Rg3 and berberine significantly decreased PA-induced green fluorescence, which indicated reduced lipid accumulation in cardiomyocytes (Figure 5F,G, *p* < 0.001). 

It was also found that H-Rg3 significantly increased gene expression of acetyl coenzyme A synthetase 2 (*Acss2*), acyl-CoA dehydrogenase (*Acadm*), diacylglycerol acyltransferase 1 (*Dgat1*), stearoyl-CoA desaturase 1 (*Scd1*), and acyl-coenzyme A oxidase 1 (*Acox1*) in the diabetic hearts following 12-week treatment, which indicated enhanced lipid metabolism (Figure 5H, *p* < 0.05). After treating with ginsenoside Rg3, the gene expressions were upregulated, including pyruvate dehydrogenase kinase 4 (*Pdk4*), glucose transporter 4 (*Glut4*), pyruvate dehydrogenase α (*Pdha*), hexokinase 2 (*Hk2*), phosphofructokinase (*PFKM*), and citric acid synthase (*Cs*), which demonstrated improved glucose metabolism (Figure 5I, *p* < 0.05). Accordingly, H-Rg3 and berberine treatment significantly increased protein levels of PPAR-γ coactivator 1α (PGC1α), CD36, carnitine palmitoyltransferase 1a (Cpt1a), Cpt1b, Cpt2, Glut4, and PDK4 in PA-incubated H9c2 cells (Figure 5J,K, *p* < 0.05). 

### 2.7. Ginsenoside Rg3 Improved Mitochondrial Function in DCM

Mitochondrial damage has been related to the disturbance of energy metabolism and several cardiovascular diseases’ occurrence [18]. Hearts of diabetic mice exhibited obvious alteration in mitochondrial morphological appearance, which indicated mitochondrial damage, but ginsenoside Rg3 and metformin increased mitochondrial size and improved mitochondrial morphological appearance (Figure S2A,B, *p* < 0.001). Furthermore, ginsenoside Rg3 and berberine increased the number of mitochondria in PA-treated cells, as evidenced by increased red fluorescent intensity (Figure S2C,D, *p* < 0.05). In addition, pretreatment with Rg3 significantly improved mitochondrial respiratory capacity suppressed by PA incubation, including maximal respiration and spare respiration (Figure S2E,F, *p* < 0.05). JC-1 staining also indicated that berberine and ginsenoside Rg3 pretreatment markedly attenuated PA-induced ΔΨm reduction (Figure S3A,B, *p* < 0.05). Correspondingly, ginsenoside Rg3 and berberine supplementation also ameliorated the expression of genes related to mitochondrial function, including Sirt1, succinate dehydrogenase enzyme subunit A (Sdha), Cyc1, ATP5j, ATP5h, Opa1, Fis1, dynamin-related protein 1 (Drp1), mitofusin 1 (Mfn1), and Mfn2 (Figure S3C, *p* < 0.05). Consistently, ginsenoside Rg3 and berberine incubation increased Mfn2 and transcription factor A mitochondrial (TFAM) protein levels, as well as decreased mitochondrial division protein levels compared to those in the model group, which indicated improved mitochondrial dynamics (Figure S3D–G, *p* < 0.01). 

### 2.8. Ginsenoside Rg3 Directly Bound to PPAR-γ and Mediated Adiponectin Pathway

**Figure 6:**
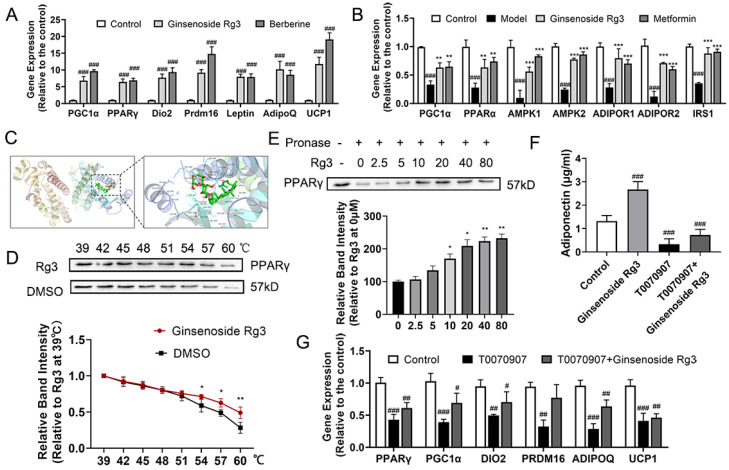
Ginsenoside Rg3-activated adiponectin pathway through directly binding to PPAR-γ. Transcription levels of genes involved in (**A**) PPAR-γ in adipocytes and (**B**) adiponectin in cardiomyocytes in four indicated groups. (**C**) Molecular docking of ginsenoside Rg3 and PPAR-γ. Representative blot images and quantitative analysis of protein levels of (**D**) CETSA and (**E**) DARTS showing the direct binding of ginsenoside Rg3 and PPAR-γ. (**F**) Adiponectin level in supernatant of cultured 3T3-L1 medium measured by ELISA kits. (**G**) Transcription levels of genes involved in PPAR-γ in adipocytes in three indicated groups. Data are expressed as the mean ± SEM (*n* = 3–6). * *p* < 0.05 or ** *p* < 0.01 or *** *p* < 0.001 vs. model group; ^###^ *p* < 0.001 or ^##^ *p* < 0.01 or ^#^ *p* < 0.05 vs. the control.

To elucidate the molecular mechanism of ginsenoside Rg3 in protecting against diabetic cardiomyopathy, gene expression related to the adiponectin pathway in adipocytes and cardiomyocytes was assessed. Treatment with both ginsenoside Rg3 and berberine induced the expression of *PPAR-γ* and adiponectin (*Adipoq*), which promoted the secretion of adipocytokines. The levels of *PPAR-α*, PPAR-γ coactivator 1α (*Pgc1α*), *Prdm16*, and uncoupling protein 1 (*Ucp1*) also increased, indicating the browning of adipocytes (Figure 6A, *p* < 0.05). Genes related to the adiponectin signaling pathway were all markedly ameliorated after treating with ginsenoside Rg3 and metformin in cardiomyocytes, including the levels of insulin receptor substrate 1 (*Irs1*), *PPARα, Pgc1α,* adenosine 5’-monophosphate (AMP)-activated protein kinase 1 (*Ampk1*), *Amk2*, adiponectin receptor 1 (*Adipor1*), and *Adipor2*, indicating an improvement in adiponectin signaling in ginsenoside Rg3-treated cardiomyocytes (Figure 6B, *p* < 0.05). 

Adiponectin is secreted by adipocytes and can be regulated by PPAR-γ. Therefore, we used molecular docking to test the binding of ginsenoside Rg3 and PPAR-γ. Ginsenoside Rg3 bound with key amino acid Ser 342, Met 364, and His 449 residues in the PPAR-γ LBD with docking score of −4.8 kcal/mol, which showed that ginsenoside Rg3 enantiomers can act on PPAR-γ (Figure 6C), so CETSA and DARTS were used to further confirm this direct interaction. PPAR-γ started to degrade at 51 °C and almost disappeared at 60 °C in vehicle-treated cells, while it degraded at 54 °C and disappeared at 60 °C in ginsenoside Rg3-treated cells (Figure 6D). After exposure to ginsenoside Rg3 for 1 h, the PPAR-γ level of the pronase E group was higher than the untreated group’s and the effect was dose dependent. (Figure 6E), which confirmed that ginsenoside Rg3 directly targeted PPAR-γ. These results were also confirmed by adding T0070907, a PPAR-γ inhibitor. After adding T0070907, secretion of adiponectin from adipocytes significantly decreased (Figure 6F), and gene levels related to browning and secretion of adiponectin were also significantly reduced (Figure 6G). Treatment with T0070907 inhibited the effects of ginsenoside Rg3, which meant the protective effects of ginsenoside Rg3 were PPAR-γ dependent.

## 3. Discussion

DCM is characterized by abnormal adipose function, which leads to decreased secretion of adiponectin and increased release of proinflammatory cytokines and free fatty acids into the serum [19]. Adiponectin directly binds to its receptors, regulating lipid and glucose metabolism in diverse organs, including the heart [20]. In this study, ginsenoside Rg3 reduced the body weight of diabetic mice by directly binding to PPAR-γ and promoting adiponectin secretion from adipocytes, thereby lowering lipid accumulation and mitochondrial dysfunction in diabetic hearts and ameliorating heart failure.

Dysfunctional white adipose tissue contributes to ectopic fat deposition in other tissues like the liver and heart, which results in insulin resistance and increased risk of cardiovascular diseases [21]. While adipogenesis is reportedly driven by PPAR-γ, it also regulates the browning of adipocytes by promoting the expression of *UcP1*, *Pgc1*, *Prdm16*, and *AdiPoq*. In this study, ginsenoside Rg3 directly bound to PPAR-γ and also promoted PPAR-γ expression in adipocytes, which enhanced the browning of adipocytes and promoted energy utilization. This may provide more evidence that Rg3 promotes energy consumption to reduce body weight. 

Rg3 treatment improved adipose function, and PPAR-γ and Adipoq expression were also increased, which promoted the secretion of adiponectin. Adiponectin can protect against HFD-induced glucose intolerance and dyslipidemia, and white adipose tissue-specific induction of adiponectin has been reported to rescue the diabetic phenotype of leptin-deficient ob/ob mice [22,23]. Therefore, Rg3 treatment promoted adiponectin secretion and alleviated insulin resistance in diabetic mice, as evidenced by reduced insulin, glucose, and free acid levels. In turn, improved hepatic function and lipid accumulation were detected.

Previous studies have demonstrated that adiponectin administration improves insulin sensitivity and glucose metabolism in the heart [24]. In this study, the expression of ADIPOR1 and ADIPOR2 was increased by ginsenoside Rg3 treatment, indicating an activated adiponectin pathway in diabetic heart. Subsequent activation of AMPK, PGC1α, PPARα, and CD36 promoted the utilization and oxidation of free acids, and increased expression of Glut4 and PDK4 indicated improved glucose utilization, and increased expression of IRS1 also indicated alleviated insulin resistance, which promoted energy production in cardiomyocytes and inhibited heart failure.

Adiponectin also improved PGC1α signaling, which activated genes related to mitochondrial biogenesis, mitochondrial dynamics, and augmentation of mitochondrial respiratory capacity, such as Drp1, Mfn, Fis1, Sirt1, Sdha, and ATP5h. While PGC1α is downregulated in the failing heart, overexpression of PGC1α in the heart can adverse the phenotypes [25,26]. Interruption of fusion/fission affects mitochondrial respiration, which is associated with the development of cardiomyopathy [27]. Increased mitochondrial fragmentation and downregulation of mitochondrial fusion and genesis proteins were found in diabetic hearts, and decreased mitochondrial respiration and membrane potential were observed. Mitochondrial dysfunction decreases energy production to preserve cardiac function, eventually leading to heart failure. However, ginsenoside Rg3 improved the expression of PGC1α, thereby alleviating mitochondrial fragmentation and improving mitochondrial respiration, which prevented heart failure in diabetic mice.

In diabetes, proinflammatory cytokines, such as TNF-α, IL-1, and IL-6, activate inflammatory signaling pathways, which lead to cardiac fibrosis, hypertrophy, cell death, systolic and diastolic dysfunction, and finally heart failure [28]. Ginsenoside Rg3 treatment improved adipose function, reduced proinflammatory cytokines, and alleviated mitochondrial dysfunction. Therefore, ginsenoside Rg3 could alleviate heart failure in diabetic mice.

However, this research also has several deficiencies. For example, ginsenoside Rg3 treatment also alleviated PA-induced H9c2 injury, which is in the absence of adiponectin. Therefore, whether the protective effect of ginsenoside Rg3 on diabetic cardiomyopathy completely depends on the adiponectin-mediated pathway remains to be further studied in conditional adipor-knockout mice. Furthermore, whether ginsenoside Rg3 improved diabetic cardiomyopathy through directly acting on the heart or through regulating adipose function can be further demonstrated by a non-obese diabetic model. Finally, ginsenoside Rg3 has been approved to be used for cancer treatment mostly in China. Clinical studies [29] and long-term safety studies [30,31] showed that ginsenoside Rg3 has good clinical effectiveness with few adverse reactions. However, the translation of our findings and the beneficial effects of ginsenoside Rg3 need to be further demonstrated in human cardiomyocytes and human patients with diabetic cardiomyopathy.

In conclusion, ginsenoside Rg3 protects against diabetic cardiomyopathy through modulating glucose and lipid metabolism by directly binding to PPAR-γ and activation of the adiponectin pathway. Our data indicate that ginsenoside Rg3 can be a promising drug in treating DCM.

## 4. Materials and Methods

### 4.1. Experimental Animals

All animal experiments were approval by the Laboratory Animal Ethics Committee of the Institute of Medicinal Plant Development, Chinese Academy of Medical Sciences (approval number: SLXD-20200113001) and conformed to the Guide for the Care and Use of Laboratory Animals published by the National Institutes of Health (NIH Publication #85–23, revised 1996). Eight-week-old male db/m mice and db/db mice were bought from Vital River Laboratory Animal Technology Co., Ltd. (Beijing, China). All animals were housed in a constant temperature room on a 12 h light–dark cycle and had free access to food and water. Mice were randomized into six groups when they were 24 weeks old: (1) db/m with water (control, n = 6); (2) db/db with water (model, n = 4); (3) db/db with metformin 250 mg/kg/day (metformin, n = 3); (4) db/db with ginsenoside Rg3 100 mg/kg/day (high dose of ginsenoside Rg3, H-Rg3, n = 6); (5) db/db with ginsenoside Rg3 50 mg/kg/day (moderate dose of ginsenoside Rg3, M-Rg3, n = 6); (6) db/db with ginsenoside Rg3 25 mg/kg/day (low dose of ginsenoside Rg3, L-Rg3, n = 6). Mice were gavaged with drugs dissolved with purified water or purified water daily for 12 weeks. Ginsenoside Rg3 with 98.179% purity came from Chengdu Must Bio-technology Co., Ltd. (Chengdu, China) and metformin was obtained from Sino American Shanghai Squibb Pharmaceutical Co., Ltd. (Shanghai, China). 

### 4.2. Blood Tests

After 12 weeks of treatment, fasting blood glucose levels were measured in overnight fasted mice using a glucometer. Blood samples were drawn retro-orbitally from mice after anesthesia, centrifuged at 3000× *g* for a quarter of an hour, and then gathered for further analysis. Free fatty acid (FFA), total triglyceride (TG), total cholesterol (CHO), high-density lipoprotein (HDL), and low-density lipoprotein (LDL), as well as alanine aminotransferase (ALT) and aspartate aminotransferase (AST), creatine kinase (CK), creatine kinase-MB (CK-MB), and lactate dehydrogenase (LDH), were all measured by an automatic biochemical analyzer in accordance with the instructions (AU480, Beckman Coulter, Brea, CA, USA). Catalase (CAT), glutathione peroxidase (GSH-px), superoxide dismutase (SOD), and malondialdehyde (MDA) assay kits were used according to the manufacturer’s instructions to assess liver function (Nanjing Jiancheng Bioengineering Institute, Nanjing, China). Kits of insulin, adiponectin, leptin, tumor necrosis factor-α (TNF-α), C-reactive protein (CRP), interleukin 1α (IL-1α), interleukin-6 (IL-6), and monocyte chemoattractant protein-1 (MCP1) bought from the Beijing Huaying Institute of Biological Technology were used to quantify serum biochemical indicators.

### 4.3. Echocardiography

At the end of treatment, echocardiography (VisualSonics, Toronto, ON, Canada) was performed by a Vevo 770 Imaging System twice. Isoflurane (RWD, Shenzhen, China) was used to anesthetize the mice. Left ventricular internal diameter (LVID), left ventricular anterior wall thickness (LVAW), and left ventricular posterior wall thickness (LVPW) were measured. Left ventricular ejection fraction (EF), fractional shortening (FS), and left ventricular (LV) volume were calculated.

### 4.4. Histology and Transmission Electron Microscopy

After formalin fixation and paraffin embedding, heart, liver, and subcutaneous adipose tissue were further used for histological analysis. Masson’s trichrome, hematoxylin and eosin (HE), and Oil Red O staining and TdT-mediated dUTP nick end labeling (TUNEL) were performed after cutting into 5 μm sections. HE and Oil Red O were used to stain mice’s livers. The adipose tissue was stained with HE. Images were then analyzed with a panoramic scanning microscope and processed using Caseviewer version 2.4 (3DHISTECH, Budapest, Hungary). Left ventricles of mouse hearts were fixed with 2.5% glutaraldehyde for transmission electron microscopic analysis (Sigma, Ronkonkoma, NY, USA). Mitochondrial ultrastructure was observed by a Tecnai G2 Spirit transmission electron microscope (Thermo Fisher Scientific, Waltham, MA, USA).

### 4.5. Cell Culture and Treatment

H9c2 cardiomyocytes were purchased from the Cell Bank of the Chinese Academy of Sciences (Shanghai, China). Cells were cultured with Dulbecco’s modified Eagle’s medium (DMEM) with 10% fetal bovine serum (FBS) at 37 °C under a 5% CO_2_ atmosphere. After cells reached approximately 80% confluence, ginsenoside Rg3 or 5 μM berberine dissolved with DMSO was added, they were cultivated for 24 h, and then palmitate acid (PA) was added or not for 24 h. 3T3-L1 cells were obtained and maintained the same to H9c2 cells. Two days after the cells reached confluence, differentiation was induced by adding 1 μM dexamethasone, 0.5 mM 3-isobutyl-1-methylxanthine, and 10 μg/mL insulin in DMEM with 10% FBS for 48 h. Two days later, the medium was replaced with a differentiation medium (10 μg/mL insulin in DMEM with 10% FBS). After 2 days’ treatment, the medium was discarded and the cells were kept for subsequent experiments. Drugs were added to 3T3-L1 cells when changing medium to induction or differentiation. Berberine with 98.38% purity was bought from Chengdu Must Bio-technology Co., Ltd. (Chengdu, China).

### 4.6. Cell Viability

Cell Counting Kit-8 (CCK-8) was used to evaluate cell viability (Solarbio, Beijing, China). Cells that were treated with ginsenoside Rg3 or PA were treated with CCK-8 for 2 h in an incubator. At the end of the treatment, a microplate reader was used to measure the absorbance at 450 nm.

### 4.7. BODIPY Staining

Lipid accumulation in cardiomyocytes and preadipocytes was evaluated by BODIPY^TM^ 493/503 (Invitrogen, Carlsbad, CA, USA). After ginsenoside Rg3 or PA treatment, cells were fixed and stained following the instructions. Images were acquired through fluorescence microscopy (Olympus, Tokyo, Japan).

### 4.8. Mitochondrion Membrane Potential (ΔΨm)

JC-1 (Thermo Fisher Scientific) was used to measure changes in ΔΨm of H9c2 cells. After treatment with 20, 10, or 5 μM ginsenoside Rg3 or 300 μM PA, the cardiomyocytes were washed with PBS and then stained with 2 μg/mL JC-1. A fluorescence microscope (Olympus) was used to image them. 

### 4.9. Mitochondrial Staining

After incubating with ginsenoside Rg3 or PA, Mitotracker Red and Hoechst 33342 were used to stain the cardiomyocytes for half an hour at 37 °C (Invitrogen) after washing. A fluorescence microscope (Olympus) was used to image the mitochondria.

### 4.10. Measurement of Mitochondrial Oxygen Consumption Rate (OCR)

An extracellular flux analyzer (XF24) was used to record H9c2 cells’ O_2_ consumption rate (OCR) according to the manufacturer’s instructions (Agilent Seahorse Bioscience, Billerica, MA, USA). Cells were seeded in cell culture microplates and, after ginsenoside Rg3 or PA treatment, OCR was recorded following the experimental protocol. Basal, maximal OCRs, ATP-related respiration, and spare respiration were calculated and all were normalized to cell counts.

### 4.11. Measurement of Adiponectin in 3T3-L1 Medium

The medium of 3T3-L1 cells was collected at the end of differentiation after centrifugation. Standard or medium was added to plates of ELISA and incubated at 37 °C (Hiton, Beijing, China) and washed 3 times, then enzyme was added and incubated at 37 °C for half an hour, after which the chromogenic agent was added and the absorbance was measured at 450 nm with a microplate reader after adding stop solution.

### 4.12. Quantitative Reverse-Transcription PCR

After 12-week treatment, hearts of mice were obtained and stored in −80 °C. The total mRNA samples were isolated from the hearts by TRIzol (Thermo Fisher Scientific), then reverse transcription was performed for qRT-PCR and the expression levels were calculated by the ^ΔΔ^Ct method.

### 4.13. Western Blotting Analysis

After treated with ginsenoside Rg3 or PA, cells were harvested and fragmented with lysis buffer at 4 °C (CWBIO, Taizhou, China). After being centrifuged and boiled, samples were loaded into each well and separated for electrophoresis. After being transferred onto nitrocellulose membranes, blocked for 2 h, and incubated with primary and secondary antibodies (Abclonal, Wuhan, China), protein bands were detected by ECL and protein levels were quantified using ImageJ. All experiments were performed at least thrice, and the average values were compared. 

### 4.14. Molecular Docking

The crystal structure of PPAR-γ (PDB ID: P37231; PDB code 6L89 with ligand E7c) was retrieved from the website https://www.rcsb.org/, on 21 January 2021. Docking was performed using AutoDockTools version 1.5.6 (the Scripps Research Institute). PyMol version 2.2.0 (Schrodinger LLC, New York, NY, USA) was used to perform the analysis and visual investigation of ligand–protein interactions of docking poses.

### 4.15. Cellular Thermal Shift Assay (CETSA)

Protein from 3T3-L1 cells was extracted using lysis buffer with 1% protease and phosphatase inhibitors, and the supernatant was subjected to the CETSA. After being incubated with ginsenoside Rg3 at 80 µM, cells were equally divided into 10 parts and heated at different temperatures (39, 42, 45, 48, 51, 54, 57, and 60 °C). Then, cell lysates were extracted by centrifugation and 5× loading buffer was added, and levels of PPAR-γ were assessed by Western blotting.

### 4.16. Drug Affinity Responsive Target Stability (DARTS)

To perform the DARTS assay, protein from adipocytes was extracted as above. The supernatant was incubated with ginsenoside Rg3 at 0, 2.5, 5, 10, 20, 40, and 80 µM, then treated with pronase (1.25 µg/mL per sample). Then, 5× loading buffer was added and boiled. Then, the samples were analyzed by Western blot.

### 4.17. Statistical Analysis

SPSS was used to perform all statistical analyses. Data are presented as the mean ± standard error of mean. One-way analysis of variance and least significant difference (LSD) analysis were used to compare multiple groups. Results were considered significantly different when *p* < 0.05.

## Data Availability

The data of this study is available from the corresponding authors upon request.

## Supplementary Materials

The supporting information can be downloaded at: https://www.mdpi.com/article/10.3390/ijms242316736/s1.

## Abbreviations

ALT, alanine aminotransferase; AST, aspartate aminotransferase; CAT, catalase; CK, creatine kinase; CK-MB, creatine kinase-MB; CRP, C-reactive protein; DCM, diabetic cardiomyopathy; EF, ejection fraction; FFA, free fatty acid; FS, fractional shortening; GSH-px, catalase glutathione peroxidase; LDH, lactate dehydrogenase; LV, left ventricular; LVAW, left ventricular anterior wall thickness; LVID, left ventricular internal diameter, LVPW, left ventricular posterior wall thickness; MCP1, monocyte chemoattractant protein-1; MDA, malondialdehyde; PA, palmitate acid.
