# Supplementary material for: Ginsenoside Rg3 Protects against Diabetic Cardiomyopathy and Promotes Adiponectin Signaling via Activation of PPAR-γ
Source: Int J Mol Sci. 2023 Nov 24;24(23):16736. doi: 10.3390/ijms242316736 (PMC10705869; doi:10.3390/ijms242316736)
Supplement: Supplementary file 1 [file ijms-24-16736-s001.zip › ijms-2702418-supplementary.pdf]

SUPPLEMENTARY FIGURES

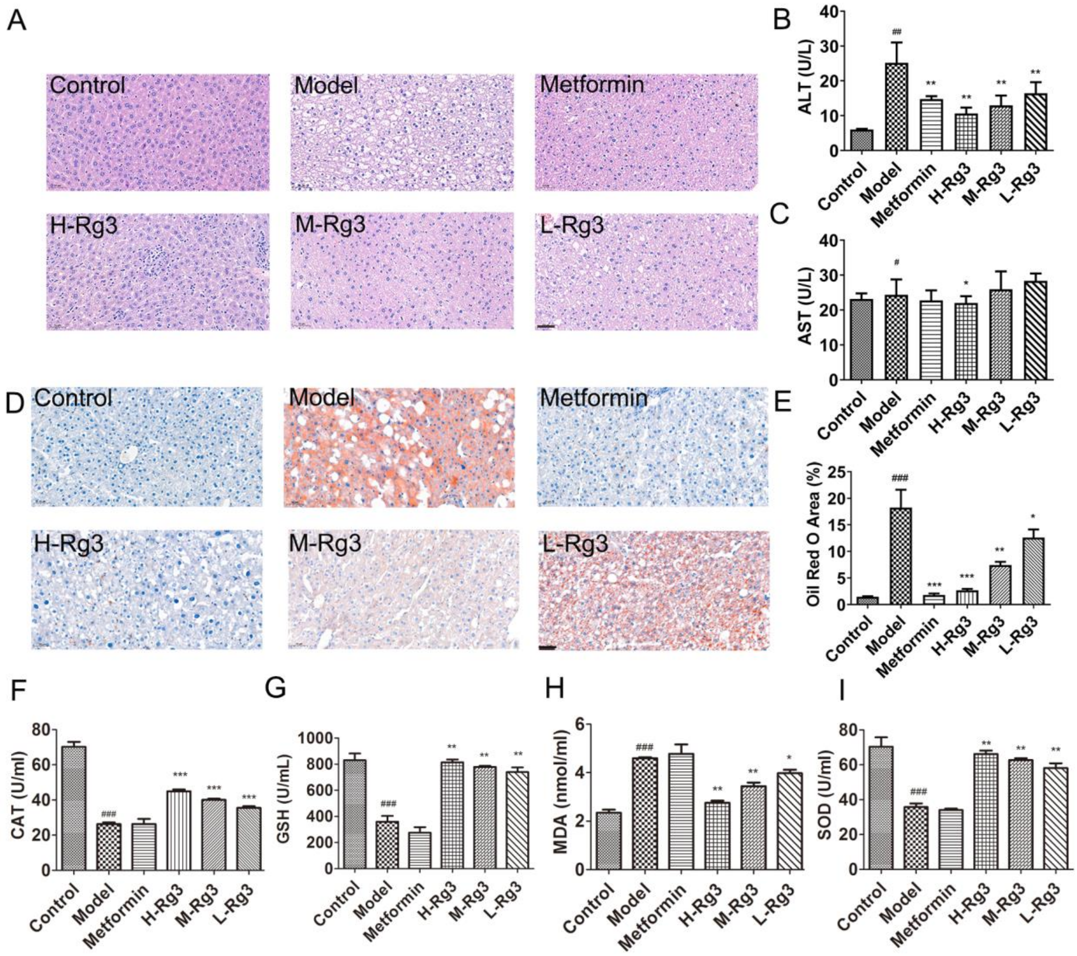

Figure S1. Ginsenoside Rg3 reduced lipid accumulation in the liver and promoted liver function. (**A**) representative images of HE staining and (**D**) representative images of Oil red O staining and (**E**) quantitative analysis of Oil red O stainings in five groups. Scale bar, 50 μm. Magnification, 200×. Serum (**B**) ALT, (**C**) AST, (**F**) CAT, (**G**) GSH-px, (**H**) MDA, and (**I**) SOD levels in different groups. Data are expressed as the mean ± SEM (n = 3-6). ***p<0.001, **p<0.01 or *p<0.05 vs. model group; $^{###}p$<0.001 or $^{##}p$<0.01 or $^{#}p$<0.05 vs. the control. H-Rg3, 100 mg/kg/day ; M-Rg3, 50 mg/kg/day; L-Rg3, 25 mg/kg/day.

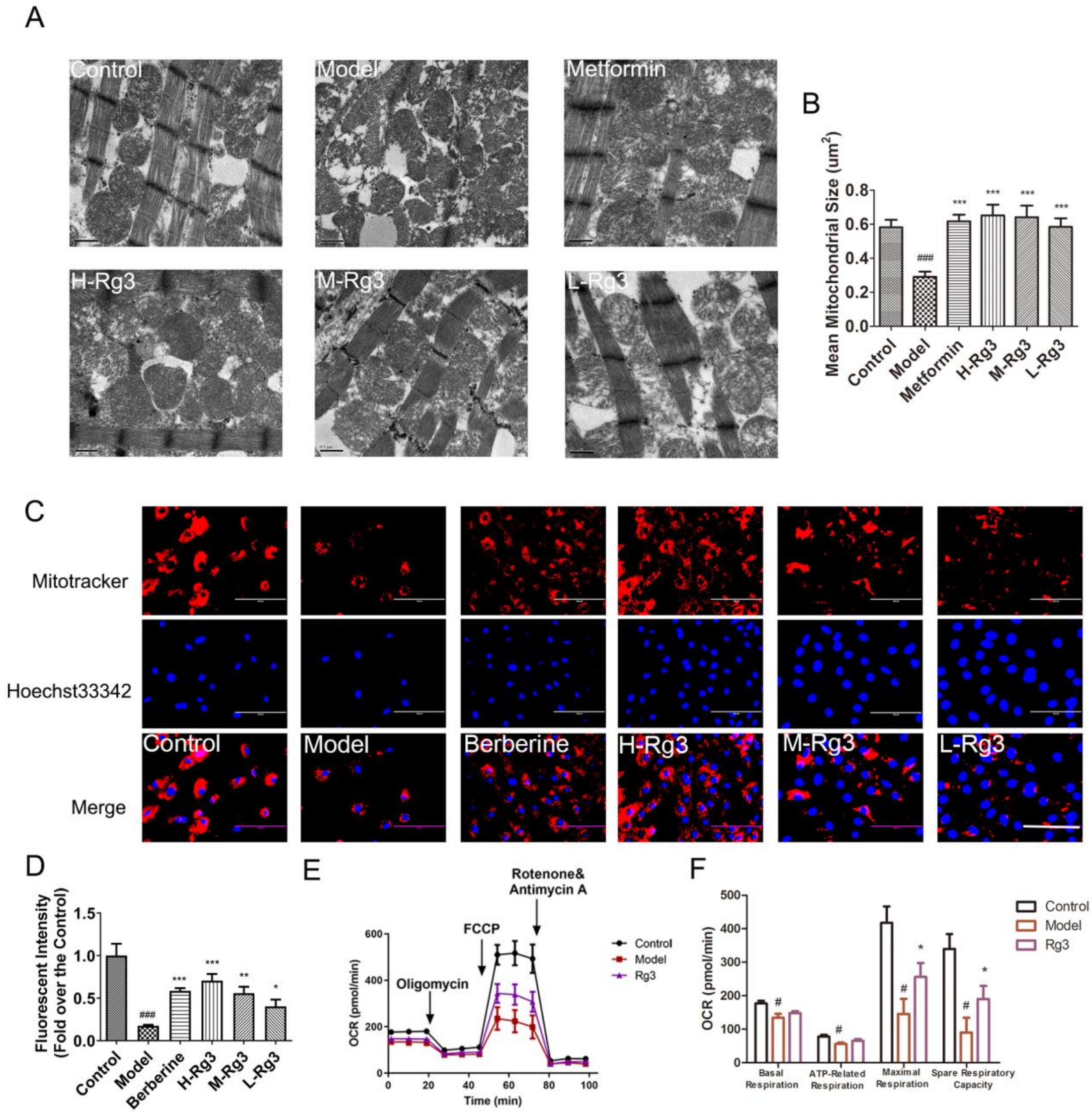

Figure S2. Ginsenoside Rg3 treatment improved myocardial mitochondrial morphology and function. (**A**) Representative transmission electron microscopy images. Scale bar, 0.5 μm. H-Rg3, 20 μM; M-Rg3, 10 μM; L-Rg3, 5 μM. (**B**) Quantitative analysis of mitochondrial size. (**C**) Representative images of mitochondria stained using MitoTracker Red. Scale bar, 100 μm. Magnification, 400×. (**D**) Quantitative analysis of mitochondrial number calculated using fluorescence intensity. (**E**) OCR and (**F**) quantitative statistical analysis of OCR. Data are expressed as the mean ± SEM (n = 3-6). $^{*}p<0.05$ or $^{**}p<0.01$ or $^{***}p<0.001$vs. model group; $^{\#}p<0.05$ or $^{\#\#\#}p<0.001$ vs. the control.

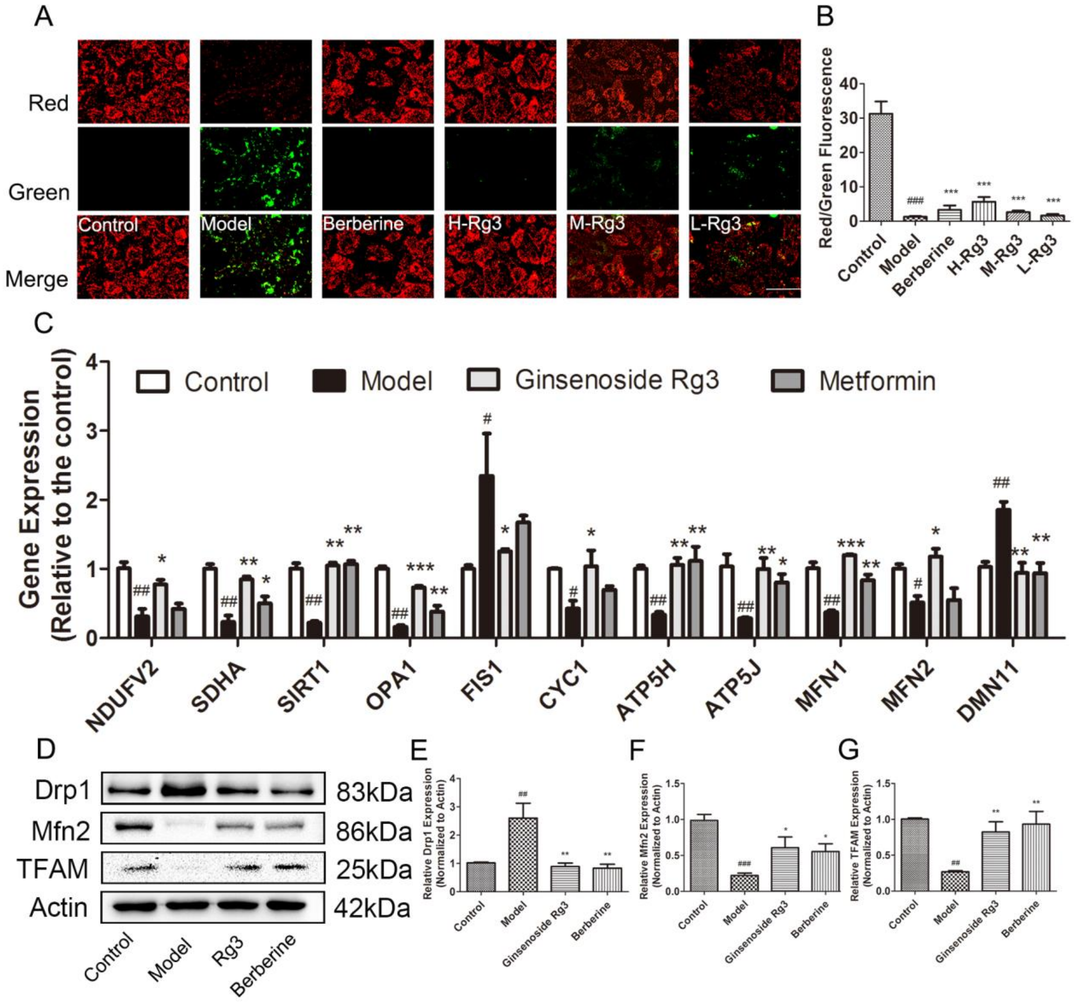

Figure S3. Ginsenoside Rg3 treatment improved myocardial mitochondrial membrane potential and dynamics. (**A**) Representative images of mitochondrial membrane potential stained using JC-1 in H9c2 cardiomyocytes. Scale bar, 100 μm. Magnification, 400×. H-Rg3, 20 μM; M-Rg3, 10 μM; L-Rg3, 5 μM. (**B**) Quantitative analysis of mitochondrial membrane potential shown by ratio of red and green fluorescence. (**C**) Transcription levels of genes related to mitochondrial respiration and dynamics in four indicated groups. (**D**) Levels of proteins related to mitochondrial biogenesis, division, and fusion estimated using western blotting. (**E**) Drp1; (**F**) Mfn2 and (**G**) TFAM protein levels in the four study groups. Data are expressed as the mean ± SEM (n = 3-6). **p*<0.05 or ***p*<0.01 or ****p*<0.001 vs. model group; $^{\#}p$<0.05 or $^{\#\#}p$<0.01 or $^{\#\#\#}p$<0.001 vs. the control.
